# Posaconazole mitigates α-amanitin-induced liver injury by inhibiting STT3B-mediated glycosylation and cellular uptake

**DOI:** 10.3389/fphar.2026.1864484

**Published:** 2026-07-10

**Authors:** Bei Wang, Chenwei Wang, Yizhi Zhang, Yingjie Yu, Jiahao Yuan, Pan Chen, Jie Chen, Yubo Tang, Qiao-Ping Wang, Ke-Jing Tang

**Affiliations:** 1 Department of Pharmacy, The First Affiliated Hospital of Sun Yat-Sen University, Guangzhou, China; 2 State Key Laboratory of Oncology in South China, Guangdong Provincial Clinical Research Center for Cancer, Sun Yat-sen University Cancer Center, Guangzhou, China; 3 Department of Liver Surgery, Sun Yat-sen University Cancer Center, Guangzhou, China; 4 School of Pharmaceutical Sciences, Sun Yat-sen University, Guangzhou, China; 5 Laboratory of Metabolism and Aging, School of Pharmaceutical Sciences, Shenzhen Campus of Sun Yat-sen University, Shenzhen, China; 6 Guangdong Provincial Key Laboratory of Diabetology, The Third Affiliated Hospital of Sun Yat-sen University, Guangzhou, China; 7 State Key Laboratory of Anti-Infective Drug Discovery and Development, School of Pharmaceutical Sciences, Sun Yat-sen University Shenzhen Campus, Shenzhen, China; 8 Division of Pulmonary and Critical Care Medicine, The First Affiliated Hospital of Sun Yat-sen University, Guangzhou, China

**Keywords:** glycosylation, liver injury, posaconazole, STT3B, α-amanitin

## Abstract

**Background:**

Mushroom poisoning caused by *Amanita phalloides*, primarily mediated by the toxin α-amanitin (AMA), frequently leads to fatal liver failure, yet no specific antidote is currently available. Although AMA is known to inhibit RNA polymerase II, its complete cytotoxic mechanism remains unclear. This study aimed to repurpose the antifungal drug posaconazole as a first-in-class antidote targeting the host glycosylation machinery to mitigate AMA-induced liver injury.

**Methods:**

Posaconazole was identified through STT3B-focused virtual screening followed by *in vitro* cell-based screening. Its protective effects were evaluated in 2D and 3D cell cultures via microscopy, cell viability assays and Calcein-AM/PI staining. Therapeutic efficacy *in vivo* was assessed in a lethal mouse model by serum biochemical analysis, H&E, immunohistochemistry, and survival analysis. The interactions between posaconazole and STT3B was examined by molecular docking and an endoplasmic reticulum-localized luciferase reporter assay. N-glycosylation level was analyzed by lectin staining. ST6GAL1 was knockdown by shRNA. The concentrations of AMA were quantified by high-performance liquid chromatography.

**Results:**

Posaconazole robustly protected human hepatocytes from AMA-induced toxicity in both 2D and 3D cultures. In a lethal mouse model, post-exposure treatment with posaconazole significantly attenuated AMA-induced liver injury and improves survival, achieving efficacy comparable to prior candidate. Mechanistically, posaconazole potentially inhibited the STT3B-mediated N-glycosylation, thereby suppressing down-stream sialylation. The genetic ablation of sialyltransferase ST6GAL1 confirmed that reduced sialylation limits cellular AMA uptake and confers resistance to toxicity.

**Conclusion:**

Our work unveils a critical glycosylation-dependent pathway for AMA toxicity and nominates posaconazole as a clinically translatable, host-directed therapeutic candidate for the treatment of lethal Amanita phalloides poisoning.

## 1 Introduction

Mushroom poisoning is a major cause of foodborne fatalities worldwide, with the death cap (*Amanita phalloides*) accounting for most fatal cases (Kayes and Ho, 2024; Li et al., 2025; Wennig et al., 2020). The paramount fatal toxin, α-amanitin (AMA), is a cyclopeptide with a human lethal dose of approximately 0.1 mg/kg (Xue et al., 2023). AMA exerts its devastating effects primarily through the irreversible inhibition of RNA polymerase II (RNAP II), leading to transcriptional arrest and subsequent apoptotic liver cell death (Garcia et al., 2015a; Ning et al., 2024). This pathological cascade culminates in acute liver failure (Wang et al., 2025), for which no specific antidote exists clinically. Current treatments are largely supportive (Garcia et al., 2015a), underscoring an urgent unmet medical need.

The pursuit of an effective antidote for AMA has been historically focused on mitigating the nuclear damage arising from RNAP II inhibition (Garcia et al., 2022; Garcia et al. 2019; Garcia et al. 2015b). However, the intricate cellular response to AMA likely extends beyond immediate transcriptional shutdown. We hypothesized that the toxin may exploit, or trigger, key post-translational modification pathways that could serve as potential therapeutic targets. To unbiasedly identify such host factors, we previously established a discovery pipeline integrating functional genomics with computational drug screening (Wang et al., 2024). A genome-wide CRISPR-Cas9 screen revealed that the oligosaccharyltransferase STT3B, a key component of the N-glycosylation machinery in the endoplasmic reticulum, is essential for AMA-induced cytotoxicity (Wang et al., 2023). This finding implicated a fundamental biochemical process, protein glycosylation, in AMA toxicity, linking a canonical nuclear toxin to an unexpected cellular pathway. We postulated that N-glycosylation may govern the expression, stability, or function of transporters that mediate AMA uptake or downstream cell death signaling. Through virtual screening, we identified several potential STT3B inhibitors and validated their protective efficacy *in vitro*. Among these, indocyanine green (ICG), an FDA-approved tricarbocyanine dye, conferred significant hepatocellular protection against AMA toxicity by inhibiting STT3B-mediated glycosylation and reducing cellular AMA uptake. Nevertheless, the short plasma half-life of ICG limited its translational potential (Zhou et al., 2025), motivating the search for pharmacokinetically superior alternatives with comparable therapeutic efficacy.

Here, we report that posaconazole, a broad-spectrum antifungal agent, acts as an effective countermeasure against AMA poisoning through a previously unrecognized glycosylation-dependent mechanism. Experimentally, posaconazole conferred substantial protection in both 2D and 3D hepatic cultures and, more importantly, in a lethal mouse model of AMA intoxication. Mechanistic studies revealed that the protective effect of posaconazole is independent of RNAP II inhibition and instead arises from suppression of protein sialylation, which limits cellular AMA uptake. These findings identify host surface glycosylation as a key determinant of toxin entry and AMA hepatotoxicity. Together, our work establishes posaconazole as a clinically translatable candidate for amatoxin poisoning and reveals host glycosylation as a central regulator of toxin-induced liver injury.

## 2 Materials and methods

### 2.1 Materials

AMA (#A4548) was purchased from APExBIO. Posaconazole (#H20213322) was kindly provided by Aosaikang Pharmaceutical Company. ICG (#H20055881) was obtained from Dandong Pharmaceutical Company.

### 2.2 Molecular docking

The detailed docking parameters and step-by-step docking procedures were described in the previous study (Wang et al., 2023; Wang et al., 2024; Zhu and Lv, 2025b). Briefly, molecular docking was performed with Smina (a fork of AutoDock Vina 1.1.2). The crystal structure of STT3B was obtained from RCSB Protein Data Bank (PDB ID: 6S7T). The binding pocket was defined by the co-crystallized dolichylphosphate ligand. The grid box dimensions were 23.25, 24.75, 24.75 Å, with the center coordinates set to (159.012, 143.831, 166.532). The native ligand (dolichylphosphate) was re-docked into the binding pocket, yielding an RMSD of 0.478 Å, confirming the reliability of the docking procedure.

### 2.3 Cell lines and cell culture

HAP1 cells (RRID: CVCL_Y019) were obtained from Horizon Discovery. HEK293T (RRID: CVCL_0063) and HepG2 (RRID: CVCL_0027) cell lines were obtained from the American Type Culture Collection. THLE-2 cell (RRID: CVCL_3803) were purchased from Wuhan Procell. HAP1 cells were cultured in Iscove’s Modified Dulbecco’s Medium (IMDM; #12440053, Gibco) supplemented with 10% Fetal Bovine Serum (FBS; #UE500, NEWZERUM) and 1% penicillin-streptomycin (#15140122, Gibco). HEK293T and HepG2 cells were cultured in Dulbecco’s modified Eagle’s medium (DMEM; # 11995065, Gibco) supplemented with 10% FBS and 1% penicillin-streptomycin. THLE-2 cells were cultured in THLE-2 Cell Specific Culture Medium (CM-0833, Wuhan Procell). All cell lines were maintained at 37 °C in a humidified atmosphere with 5% CO_2_, routinely confirmed to be mycoplasma-free, and authenticated by short tandem repeat profiling.

### 2.4 Cell viability assay

Trypsinized cells (3–5 × 10^3^) were seeded in 96-well plate (100 μL per well) and incubated for 24 h. Compounds were then added, and cells were further cultured for an additional 48 or 72 h. Subsequently, the medium was replaced with 100 μL fresh medium containing a 10% Cell Counting Kit-8 (CCK-8; #K1018, APExBIO) and incubated for 2 h at 37 °C. Absorbance was measured at 450 nm using a microplate spectrophotometer (BioTek). Cell viability was normalized to vehicle-treated controls and expressed as relative cell viability. Cell death was additionally assessed using Calcein-AM/PI Cell Viability Assay (#C2015M, Beyotime) according to the manufacturer’s instructions and visualized under a fluorescence microscope (Nikon).

### 2.5 3D cell culture

Trypsinized cells (3–5 × 10^3^) were seeded in 96-well ultra-low adhesion cell spheroid culture plate (#AUT042802, Anchesen) in 100 μL of medium. After 24 h, compounds were added, and the spheroids were incubated for an additional 72 h before imaging under a microscope (Nikon).

### 2.6 Luciferase reporter assay

The ER-LucT sequence and LucT sequence from our previous study (Wang et al., 2023) were synthesized and cloned into pcDNA3.1 (+) by Kidan Bio Co. Ltd. Plasmid transfections were performed using Lipo8000TM (#C0533, Beyotime) according to the manufacturer’s instructions. After 48 h of transfection, posaconazole was added. Luciferase activity was measured using the Firefly Luciferase Reporter Gene Assay Kit (#RG005, Beyotime) and quantified with the Varioskan Lux (Thermo Fisher Scientific).

### 2.7 Mice

All experimental procedures involving animals were approved by the Institutional Animal Care and Use Committee (IACUC) of Sun Yat-Sen University (approval number: SYSU-IACUC-2025–001050). Male CD-1 mice (20–30 g) were obtained from the Laboratory Animal Center of Sun Yat-Sen University. Mice were maintained in a specific pathogen-free facility under controlled conditions (temperature: 23 °C ± 2 °C, humidity: 50%–65%, 12 h/12 h light/dark cycle) with *ad libitum* access to food and water. Animal wellbeing was monitored daily, including assessments of body weight, motor activity, and respiratory distress. Euthanasia was performed by intraperitoneal injection of 50 mg/kg pentobarbital sodium, followed by cervical dislocation, in accordance with IACUC-approved protocols. The study was conducted in compliance with the ARRIVE guidelines 2.0.

### 2.8 Short-term toxicity study (24 h)

Mice were randomly assigned to five experimental groups (n = 6): (1) control group: 0.9% NaCl; (2) posaconazole group: 10 mg/kg posaconazole, i. v. At 4 and 12 h; (3) AMA group: 0.33 mg/kg AMA i. p. At 0 h; (4) posaconazole + AMA: 0.33 mg/kg AMA (i.p. At 0 h) followed by 10 mg/kg posaconazole (i.v. At 4 and 12 h); (5) AMA + ICG group: 0.33 mg/kg AMA (i. p. At 0 h) followed by 5 mg/kg ICG (i.v. At 4, 6, and 8 h). Sample sizes were determined based on previous experience with the AMA-induced liver injury model (Wang et al., 2023) and comparable published studies (Garcia et al., 2019; Xu H. et al., 2025).

### 2.9 Analysis of blood biomarkers

After 24 h of the AMA administration, mice were anesthetized and euthanized. Blood was collected in EDTA-coated tubes and centrifuged at 3000 g for 10 min (4 °C). The plasma supernatant was collected and stored at −80 °C until analysis. AST, ALT, ALP, TBIL, Urea and Cre were quantified by Servicebio.

### 2.10 Histological analysis

After blood collection, livers were excised and weighed. Liver segments were fixed in 4% paraformaldehyde for 24 h, embedded in paraffin, sectioned, and stained with H&E. The degree of necrosis was semi-quantitatively assessed in a blinded manner using the following grading scale: Grade 0 = no pathological changes; Grade 1 = minimal necrosis; Grade 2 = mild necrosis; Grade3 = moderate necrosis; Grade 4 = severe, widespread necrosis.

### 2.11 Immunohistochemistry

Liver sections were deparaffinized and subjected to antigen retrieval. Endogenous peroxidase activity was suppressed by incubation with 3%H_2_O_2_ for 30 min. Non-specific binding sites were blocked with 3% BSA. Sections were then incubated overnight at 4 °C with the following specific primary antibodies: anti-F4/80 (1:1000; #GB113373, Servicebio) and Ly6G (1:1000; #GB11229, Servicebio), both of which have been validated in previous publications (Liu et al., 2025; Zhao Q. H. et al., 2025). After washing, sections were incubated for 1 h at room temperature with horseradish peroxidase (HRP)-conjugated secondary antibody (1:200; #GB23303, Servicebio). Immunoreactivity was visualized using diaminobenzidine (DAB). Staining images were quantified using ImageJ software (Version 1.54 g). F4/80 staining was quantified based on the positively stained area. After background subtraction by threshold adjustment to exclude non-specific signals, the positive area was measured as a percentage of the total field area. Ly6G staining was quantified based on the number of positive cells. After background subtraction by threshold adjustment, positive cells were counted manually.

### 2.12 Lectin staining

For lectin staining, liver sections were deparaffinized and subjected to antigen retrieval. Sections were then incubated for 30 min at room temperature with the following fluorescent lectins diluted in PBS: Cy5-SNA (1:200; #CL-1305–1, Vector Laboratories) and Fluorescein-AAL (1:200; #FL-1391–1, Vector Laboratories). Following incubation, slides were washed, and nuclei were counterstained with DAPI. Sections were mounted and imaged using a Nikon fluorescence microscope. Fluorescence intensity was quantified using ImageJ software.

### 2.13 Long-term survival and toxicity study (30 days)

For the long-term study (30-day observation), mice were randomly assigned to the same five groups (n = 6) and received an identical dosing regimen to the short-term study. Animal wellbeing was monitored daily throughout the 30-day period, including assessments of body weight, motor activity, and respiratory status.

### 2.14 Generation of ST6GAL1-knockdown cells

The shRNA sequences targeting ST6GAL1 were as previous described (Supplementary Table S1) (Gc et al., 2022). Lentiviral particles for ST6GAL1 knockdown were produced in HEK293T cells. For lentiviral production, HEK293T cells at 60% confluency in a T75 flask were co-transfected using Lipo8000™ (#C0533, Beyotime) with the shRNA plasmid, along with the packaging plasmids psPAX2 (#12260, Addgene) and pMD2. G (#12259, Addgene). After 8 h, the medium was replaced with DMEM supplemented with 10% fetal bovine serum (FBS) and 1% penicillin-streptomycin. Viral supernatant was collected at 48 and 72 h post-transfection, pooled, and filtered through a 0.45 μm low-protein-binding filter (Merck Millipore). The filtered viral supernatant was aliquoted and stored at −80 °C.

For lentiviral transduction and selection, HAP1 cells were seeded in a 6-well plate. Upon reaching approximately 50%–60% confluency, cells were transduced with lentiviral supernatant containing 8 μg/mL polybrene. After 48 h, 1 μg/mL puromycin for selection was added. Puromycin selection was maintained for 7 days, and all wild-type HAP1 cells will dead in 3 days The resulting puromycin-resistant, ST6GAL1-knockdown cells were expanded for subsequent experiments.

### 2.15 RNA extraction and quantitative real-time PCR analysis

Total RNA was extracted using an RNA Quick Purification kit (#RN001, ESscience). The cDNA was synthesized from 500 ng of total RNA by PrimeScript RT Master Mix (#RR036A, Takara) according to the manufacturer’s instructions. Quantitative PCR (qPCR) was performed with TB green (#RR820A, Takara) according to the manufacturer’s protocol with LightCycler96 (Roche). The relative expression of target genes was calculated using the 2 (-ΔΔCt) method and normalized to internal control.

### 2.16 High-performance liquid chromatography (HPLC)

A total of 2 × 10^7^ AMA-treated cells were harvested and broken via ultrasonication for 1 min on ice water. This mixture was centrifuged at 15000 g for 30 min. The supernatant was collected, mixed with 3 volumes of isopropanol, and centrifuged again to precipitate proteins. The supernatant was then passed through a 0.22 μm filter (Merck Millipore). A 30 μL aliquot of the processed sample was injected into the HPLC system equipped with a 250 mm × 4.6 mm liquid chromatography column (5 μm, Phenomenex). Chromatographic separation was achieved using a mobile phase composed of 50 mM ammonium acetate, acetonitrile, and methanol (80:10:10, v/v/v), as previously described (Kaya et al., 2014). The flow rate was maintained at 1 mL/min and analytics were detected at 303 nm. Under these conditions, the retention time of AMA was approximately 7 min. Peak areas were integrated and quantified using LabSolutions software LabSolutions software (Version 1.26). The peak area of shST6GAL1 cells was normalized to shNT control cells and expressed as the relative peak area.

### 2.17 Statistical analysis and reproducibility

Data are presented as the mean ± standard deviation (S.D.). Statistical analyses were performed using GraphPad Prism nine software (Version 10.4.1) (Zhu and Lv, 2025a; Zhu and Lv, 2025c). Specific statistical tests used for each experiment are detailed in the corresponding figure legends. For all analyses, *p* < 0.05 was considered statistically significant. The significance levels are denoted as follows: ^*^ *p* < 0.05; ^**^ *p* < 0.01; ^***^ *p* < 0.001; ^****^ *p* < 0.0001; ns, not significant. Biological replicates were stated in the legends. All experiments presented as representative micrographs were repeated at least 3 times with similar results.

## 3 Results

### 3.1 Posaconazole mitigates α-amanitin-induced hepatotoxicity *in vitro*

**FIGURE 1:**
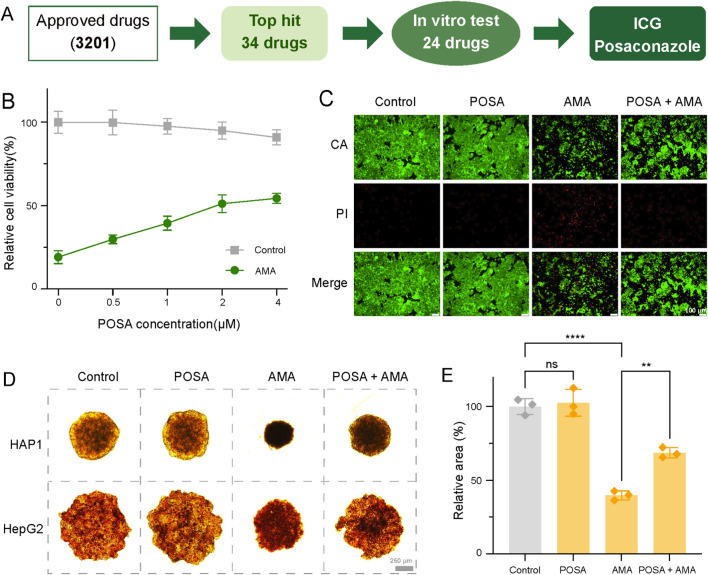
Posaconazole alleviates AMA cytotoxicity in 2D and 3D cell culture models **(A)** Schematic design of *in silico* screen to identify ICG and posaconazole (POSA) as STT3B inhibitors. Minimized affinity and NNScore2 were used as selection criterion for 34 top hits and 24 compounds were tested for the final *in vitro* test **(B)** Posaconazole blocked AMA toxicity. HAP1 cells were treated with posaconazole and AMA (3 μM) for 72 h (n = 6 biological replicates) **(C)** Calcein-AM/PI viability assay of HAP1 cells treated with posaconazole and AMA (3 μM) for 72 h, scale bars are 100 μm **(D)** Representative images of cell spheroids, scale bars are 250 μm **(E)** Relative cross-sectional area of HAP1 cell spheroids measured by ImageJ (n = 3 biological replicates). ^ns^ *p* = 0.9477, ^****^ *p* < 0.0001, ^**^ *p* = 0.0013. The statistics were assessed using one-way ANOVA followed by Tukey’s multiple comparisons test.

We previously identified ICG and posaconazole as potential STT3B inhibitors (Figure 1A). Since ICG has been characterized previously (Wang et al., 2023), in this study, we focused on posaconazole, a broad-spectrum triazole antifungal agent with established clinical use and a favorable pharmacokinetic profile (Cao et al., 2024; Maertens et al., 2021), which suggests immediate translational potential for repurposing. We first quantified the protective efficacy of posaconazole in a 2D cell model. Posaconazole treatment conferred dose-dependent amelioration against AMA-induced cell death, rescuing relative cell viability from approximately 20% in the AMA-only group to over 50% at the highest concentration tested (Figure 1B). Crucially, no statistical difference was observed between the posaconazole-alone groups and the control (0 µM posaconazole) group (all *p* > 0.05), demonstrating that posaconazole alone did not exhibit intrinsic cytotoxicity at the tested concentrations. This protective effect was further confirmed by Calcein-Acetoxymethyl Ester/Propidium Iodide (Calcein-AM/PI) staining (Figure 1C).

Given the limitations of 2D monocultures in recapitulating tissue-level physiology, we advanced our validation to a 3D spheroid model, which mimics the complex cell-cell interactions and metabolic gradients found in the liver. (Povero et al., 2023). Consistent with the 2D findings, posaconazole treatment conferred significant protection in 3D spheroids exposed to AMA. Morphometric analysis revealed that posaconazole-treated spheroids maintained significantly larger cross-sectional areas than their vehicle controls (Figures 1D,E), indicating a suppression of contraction typically associated with toxin-induced spheroid disintegration. Collectively, these findings demonstrate that posaconazole effectively mitigates AMA-induced cytotoxicity across both conventional and physiologically relevant human hepatic culture systems.

### 3.2 Posaconazole impairs STT3B-mediated N-glycosylation

**FIGURE 2:**
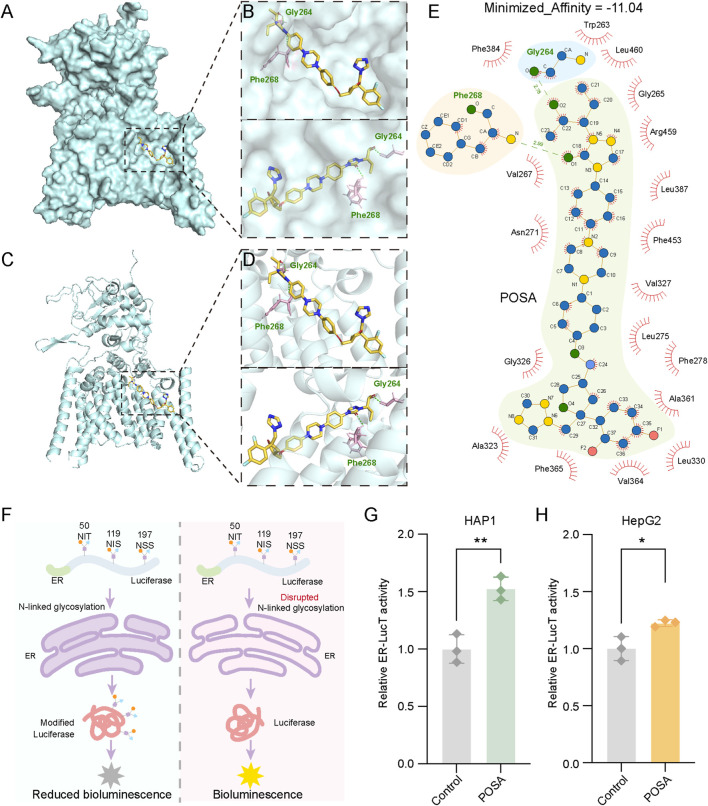
Posaconazole impairs STT3B-mediated N-glycosylation **(A)** 3D Overview and **(B)** close-up views of binding sites of STT3B and posaconazole in surface model **(C)** 3D Overview and **(D)** close-up views of binding sites of STT3B and posaconazole in cartoon model (generated by PyMOL). Posaconazole is shown in orange. STT3B residues interacting with posaconazole are shown in purple. Hydrogen bonds are shown in dotted green lines **(E)** 2D STT3B and posaconazole interaction diagrams (generated by LigPlot). Posaconazole is surrounded by a light green background, two hydrogen-bonding residues are shown in light blue and light orange, and hydrogen bonds are shown in green dotted lines, the red “eyelash”-like markers indicate the hydrophobic interactions between posaconazole and STT3B **(F)** A scheme of ER-LucT reporter system, the disruption of luciferase glycosylation turn on luminescence. The relative ER-LucT activity of control and posaconazole group in HAP1 cells **(G)** and HepG2 cells **(H)** (n = 3 biological replicates) **(G)** ^**^ *p* = 0.0048 **(H)** ^*^ *p* = 0.0236. The statistics were assessed using unpaired t-test.

To further elucidate how posaconazole inhibits STT3B activity, we performed molecular docking analysis. The results indicated that posaconazole binds stably within the substrate-binding pocket of STT3B (PDB ID: 6S7T) (Ramírez et al., 2019) with a high-affinity binding energy of −11.04 kcal/mol (Figures 2A,C). A close-up view revealed that posaconazole inserts into the pocket of STT3B, with its oxygen-containing side chain and the 1,2,4-triazole ring forming critical hydrogen bonds with Gly264 and Phe268, respectively (Figures 2B,D). The extensive hydrophobic contacts with surrounding residues further stabilized the complex (Figure 2E), collectively suggesting that posaconazole acts as a potential inhibitor by sterically occluding the catalytic site.

We next asked whether the predicted binding of posaconazole translates into functional inhibition of STT3B-mediated N-glycosylation in cells. To this end, we employed an endoplasmic reticulum (ER)-localized luciferase reporter (ER-LucT) system. This construct consists of a modified luciferase (Luc) with three (T) potential glycosylation sites fused to an ER translation sequence (Phoomak et al., 2023; Rinis et al., 2018). In this system, the N-glycosylation inhibits luciferase activity, thereby reducing bioluminescence, whereas impaired N-glycosylation increases bioluminescence (Figure 2F). We first tested the reporter by comparing LucT and ER-LucT constructs. Luminescence was significantly reduced in both HAP1 and HepG2 cells expressing the ER-targeted construct, confirming that ER-associated N-glycosylation negatively regulates luciferase activity (Supplementary Figure S1). Consistent with our docking results, posaconazole treatment elicited a marked elevation of luminescence signal in ER-LucT expressing HAP1 (Figure 2G) and HepG2 cells (Figure 2H), indicating that the drug suppresses N-glycosylation. Together, these computational and functional evidence demonstrates that posaconazole is a potential inhibitor of STT3B. The compound binds with high affinity to the enzyme’s catalytic pocket, thereby impairing N-glycosylation that is required for AMA-induced cytotoxicity.

### 3.3 Posaconazole confers hepatoprotection and improves survival in a murine model of α-amanitin poisoning

**FIGURE 3:**
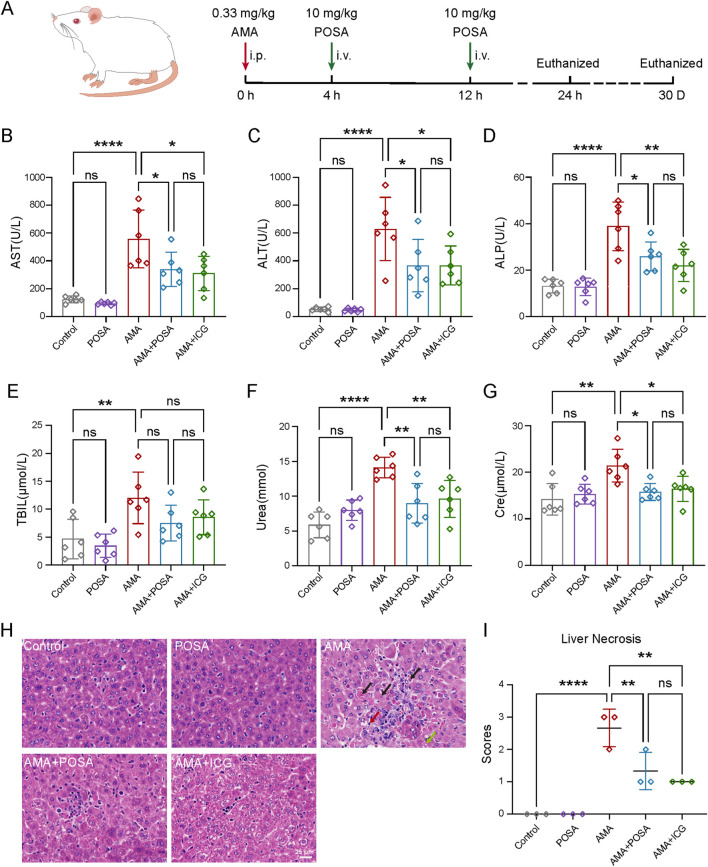
Posaconazole relieves the AMA poisoning symptoms in CD-1 mice **(A)** Diagram of drug administration in animal experiments. 0.33 mg/kg AMA was **(I)**. p. Injected at 0 h and 10 mg/kg posaconazole **(I)**. v. Injected at 4 h and 12 h **(B–G)** Plasma levels of AST, ALT, ALP, TBIL, Urea and Cre in different groups (n = 6 biological replicates) **(B)** ^ns^ *p* = 0.9928, ^****^ *p* < 0.0001, ^*^ *p* = 0.0358, ^*^ *p* = 0.0138, ^ns^ *p* = 0.9935 **(C)** ^ns^ *p* > 0.9999, ^****^ *p* < 0.0001, ^*^ *p* = 0.0328, ^*^ *p* = 0.0338, ^ns^ *p* > 0.9999 **(D)** ^ns^ *p* > 0.9999, ^****^ *p* < 0.0001, ^*^ *p* = 0.0179, ^**^ *p* = 0.0014, ^ns^ *p* = 0.8348 **(E)** ^ns^ *p* = 0.9711, ^**^ *p* = 0.0080, ^ns^ *p* = 0.1815, ^ns^ *p* = 0.4221, ^ns^ *p* = 0.9825 **(F)** ^ns^ *p* = 0.4488, ^****^ *p* < 0.0001, ^**^ *p* = 0.0027, ^**^ *p* = 0.0095, ^ns^ *p* = 0.9861 **(G)** ^ns^ *p* = 0.9570, ^**^ *p* = 0.0012, ^*^ *p* = 0.0136, ^*^ *p* = 0.0341, ^ns^ *p* = 0.9944 **(H)** Representative images of H&E staining for mice livers. Hepatocyte necrosis (black arrows), lymphocyte infiltration (red arrows), vacuolar degeneration (green arrows), scale bar is 25 μm **(I)** Liver necrosis score in different treatments (n = 3 biological replicates). ^****^ *p* < 0.0001, ^**^ *p* = 0.0082, ^**^ *p* = 0.0017, ^ns^ *p* = 0.7940. The statistics were assessed using one-way ANOVA followed by Tukey’s multiple comparisons test.

We next evaluated the efficacy of posaconazole as a potential antidote *in vivo*. We employed a well-characterized murine model of AMA intoxication, wherein CD-1 mice received an intraperitoneal (i.p.) injection of a lethal dose of AMA (0.33 mg/kg) (Garcia et al., 2019; Wang et al., 2023; Wu et al., 2022). Posaconazole was given intravenously to ensure rapid and consistent systemic delivery. To model delayed clinical intervention between ingestion and treatment, and ensure adequate drug exposure during acute intoxication, posaconazole (10 mg/kg) was administered twice at an 8-h interval, with the first dose given 4 h after AMA challenge (Figure 3A) (Chen et al., 2023; Pramanik et al., 2025; Zhao Q. et al., 2025). Because of its demonstrated efficacy against AMA toxicity, ICG was used as a positive control in animal studies (Wang et al., 2023).

As the liver is the primary target of AMA, we first examined serum biochemical markers of hepatic injury. AMA intoxication provoked severe liver damage, as evidenced by dramatic elevations in circulating aspartate aminotransferase (AST), alanine aminotransferase (ALT), and alkaline phosphatase (ALP). Posaconazole treatment significantly attenuated the increase of all three enzymes, indicating potent hepatoprotection. Notably, the efficacy of posaconazole was comparable to that of ICG (Figures 3B–D). Total bilirubin (TBIL) levels also showed a decreasing trend following treatment of posaconazole and ICG (Figure 3E). Consistently, analysis of renal function parameters revealed a congruent protective effect, with posaconazole and ICG both significantly reducing the AMA-induced increases in Urea and creatinine (Cre) (Figures 3F,G).

**FIGURE 4:**
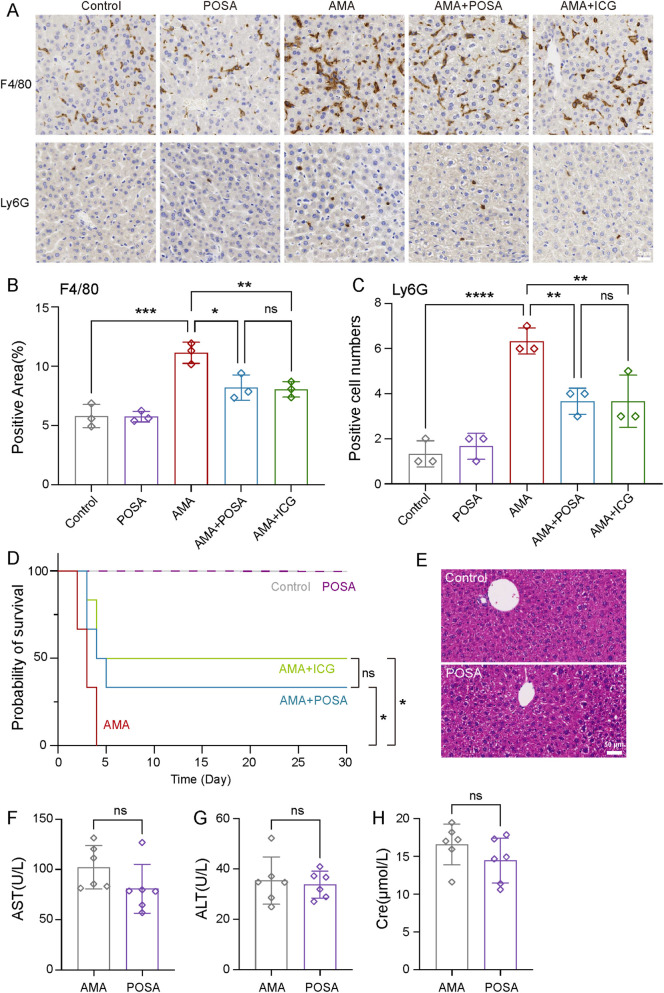
Posaconazole reduced inflammatory recruitment, improves survival rate and demonstrates safety in mice **(A)** Immunohistochemistry images of F4/80 staining (top) and Ly6G staining (bottom), scale bars are 25 μm **(B)** Quantitative analysis of positive F4/80 area. ^***^ *p* = 0.0001, ^*^ *p* = 0.0112, ^**^ *p* = 0.0082, ^ns^ *p* = 0.9995 **(C)** Quantitative analysis of positive Ly6G cell numbers. ^****^ *p* < 0.0001, ^**^ *p* = 0.0082, ^**^ *p* = 0.0082, ^ns^ *p* > 0.9999. The statistics were assessed using one-way ANOVA followed by Tukey’s multiple comparisons test **(D)** The survival curve of mice (n = 6 biological replicates). ^ns^ *p* = 0.5757, ^*^ *p* = 0.0496, ^*^ *p* = 0.0259. The statistics were assessed using survival curve comparison followed by Log-rank test **(E)** Representative images of H&E staining for mice livers after 30 days, scale bar is 50 μm **(F–H)** Plasma levels of AST, ALT and Cre for different groups in long-term study (n = 6 biological replicates) **(F)** ^ns^ *p* = 0.1387 **(G)** ^ns^ *p* = 0.7259 **(H)** ^ns^ *p* = 0.2244. The statistics were assessed using unpaired t-test.

We next performed histopathological examination of liver tissues to corroborate the biochemical findings. Hematoxylin and eosin (H&E) staining of livers from AMA-intoxicated mice revealed extensive confluent necrosis, a hallmark of AMA-induced hepatic failure. In contrast, livers from mice treated with either posaconazole or ICG displayed markedly reduced necrotic regions (Figures 3H,I). Given the intimate link between hepatocyte death and sterile inflammation, we next investigated the hepatic immune response. Immunohistochemical staining for the macrophage marker F4/80 and the neutrophil marker Ly6G showed massive infiltration of these innate immune cells following AMA exposure. Both posaconazole and ICG treatment substantially reduced this inflammatory recruitment (Figures 4A–C), indicating that suppression of primary hepatocyte death dampens the subsequent inflammatory cascade.

We further evaluate the long-term survival benefit of posaconazole in the murine model of AMA intoxication. In the absence of treatment, AMA exposure was uniformly fatal, with all mice succumbing within 96 h. In contrast, posaconazole administration following AMA challenge conferred a marked survival advantage over AMA alone (log-rank test, exact *p* = 0.0496; hazard ratio = 0.2007), rescuing animals throughout the 30-day observation period (Figure 4D). This protective effect was comparable to that of ICG treatment ((log-rank test, exact *p* = 0.0259; hazard ratio = 0.2955). Notably, no significant difference was observed between posaconazole and ICG treatment (log-rank test, exact *p* = 0.5757; hazard ratio = 0.6818). Importantly, posaconazole-treated mice showed no obvious adverse effects in long-term studies, as assessed by H&E staining (Figure 4E) and serum biomarkers (Figures 4F–H). Those findings further support the safety of this dosing regimen.

In summary, posaconazole administration, even when initiated after the onset of toxicity, effectively mitigates AMA-induced multiorgan injury, preserves hepatic architecture, and attenuates the accompanying inflammatory response *in vivo*. This intervention markedly improves survival, achieving a protective efficacy comparable to that of our previously identified lead compound ICG.

### 3.4 Posaconazole alters the hepatic N-glycosylation patterns by attenuating protein sialylation

**FIGURE 5:**
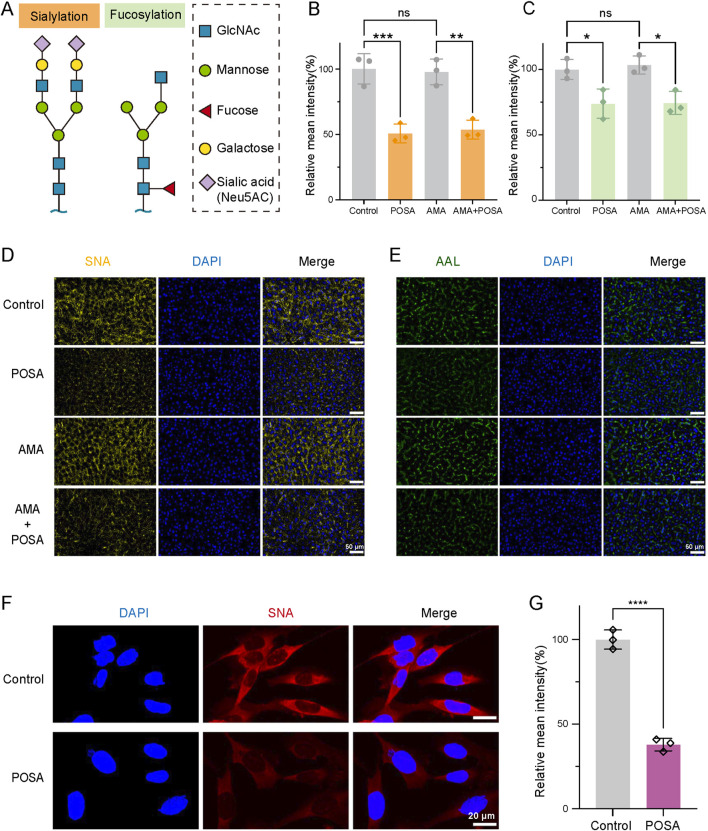
Posaconazole alters the hepatic N-glycosylation patterns **(A)** Structure diagram of sialylation and fucosylation. Semi-quantitative evaluation of Cy5-SNA **(B)** and Fluorescein-AAL **(C)** by ImageJ (n = 3 biological replicates) **(B)** ^***^ *p* = 0.0008, ^ns^ *p* = 0.9893, ^**^ *p* = 0.0017 **(C)** ^*^ *p* = 0.0275, ^ns^ *p* = 0.9648, ^*^ *p* = 0.0159. The statistics were assessed using one-way ANOVA followed by Tukey’s multiple comparisons test. Representative images of SNA **(D)** binding to sialylated glycans and AAL **(E)** binding to fucosylated glycans, scale bars are 50 μm **(F)** Representative images of SNA in control group and posaconazole treated group in THLE-2 cells, scale bars are 20 μm **(G)** Semi-quantitative evaluation of Cy5-SNA by ImageJ in control group and posaconazole treated group in THLE-2 cells (n = 3 biological replicates), ^****^ *p* < 0.0001, the statistics were assessed using unpaired t-test.

We next sought to delineate the specific impact of this inhibition on the hepatic glycoproteome *in vivo*. Given that terminal glycan modifications, such as sialylation and fucosylation, are critical for glycoprotein stability and function (Pinho et al., 2023), we profiled these epitopes in liver tissues from our murine model (Figure 5A). Lectin histochemistry using Cy5-labeled Sambucus nigra lectin (SNA), which recognizes sialylated glycans (Fan et al., 2023; Gc et al., 2022), revealed a profound reduction in sialylation signals in the livers of posaconazole-treated mice compared to controls (Figures 5B,D). Similarly, staining for core fucosylation with fluorescein-labeled Aleuria aurantia lectin (AAL) (Pan et al., 2024) was also significantly diminished (Figures 5C,E). These results confirm that pharmacological inhibition of STT3B by posaconazole effectively disrupts protein glycosylation patterns within the liver. As the effect on sialylation was particularly striking, we focused subsequent mechanistic studies on this terminal modification.

Sialylation, the addition of sialic acid (SA) residues to terminal glycan structures, is a widespread modification found on many mature glycoproteins (Ma et al., 2024; Yao et al., 2022). Mounting evidence indicates that sialylation is essential for diverse cellular processes and molecular recognition events, including host-commensal homeostasis (Yao et al., 2022), cancer progression (Fan et al., 2023; Gc et al., 2022), antitumor immunity (Wu et al., 2023) and osteoclast formation (Dou et al., 2022). To corroborate our *in vivo* observations and probe the cellular consequences directly, we next assessed sialylation levels *in vitro*. Consistent with our animal data, Cy5-SNA staining of THLE-2 cells demonstrated a significant decrease in sialylation following posaconazole treatment (Figures 5F,G). Collectively, these findings establish that posaconazole changes the N-glycosylation by significantly attenuating protein sialylation, both in cultured hepatocytes and in the intact liver.

### 3.5 ST6GAL1-mediated sialylation facilitates AMA cellular entry and cytotoxicity

**FIGURE 6:**
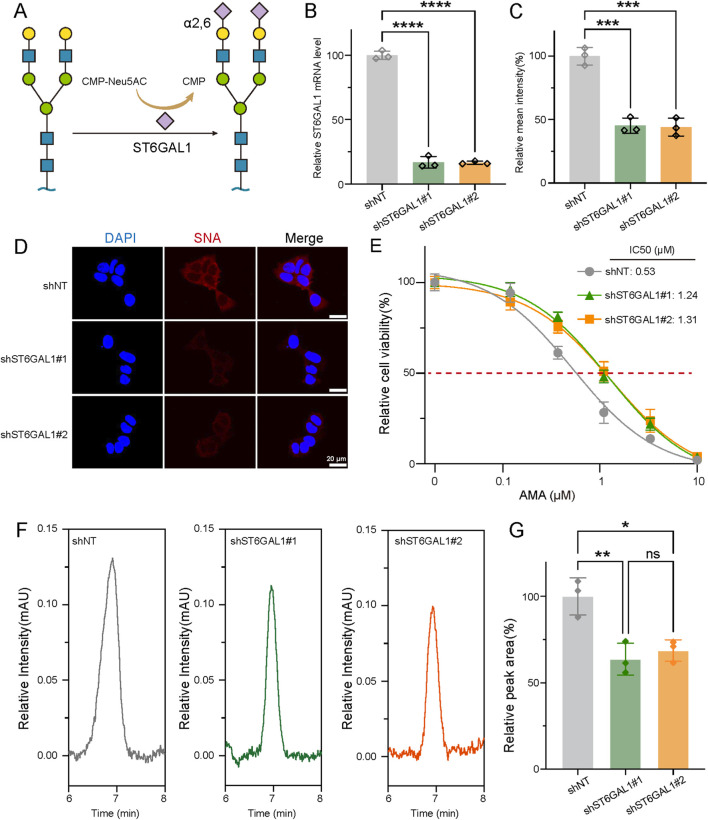
ST6GAL1-mediated sialylation facilitates AMA cellular entry and cytotoxicity **(A)** Catalytic reaction of ST6GAL1: ST6GAL1 specifically catalyzes the addition of sialic acid to Gal-*β*1,4GLcNAc via *α*2,6-linkage **(B)** Relative ST6GAL1 mRNA level in ST6GAL1 knockdown HAP1 cells (n = 3 biological replicates). ^****^ *p* < 0.0001, ^****^ *p* < 0.0001 **(C)** Semi-quantitative evaluation of Cy5-SNA by ImageJ in ST6GAL1 knockdown HAP1 cells (n = 3 biological replicates). ^***^ *p* = 0.0001, ^***^ *p* = 0.0001 **(D)** Representative images of SNA in ST6GAL1 knockdown HAP1 cells, scale bars are 20 μm **(E)** ShNT and shST6GAL1 HAP1 cells were treated with vehicle or AMA for 72 h, and cell viability was determined by CCK8 assay (n = 3 biological replicates) **(F)** The representative peak of AMA in ST6GAL1 knockdown HAP1 cells **(G)** The relative peak area of AMA in ST6GAL1 knockdown HAP1 cells (n = 3 biological replicates). ^**^ *p* = 0.0061, ^*^ *p* = 0.0123, ^ns^ *p* = 0.7860. The statistics were assessed using one-way ANOVA followed by Tukey’s multiple comparisons test.

Given that posaconazole markedly reduces sialylation, we next investigated whether this specific glycan modification functionally contributes AMA-induced cell death. We hypothesized that cell surface sialylation, catalyzed by specific sialyltransferases, might be critical for the cytotoxic action of AMA. Among these, we focused on ST6GAL1, the primary enzyme responsible for adding α2,6-linked sialic acids to N-glycans (Figure 6A) (Fan et al., 2023), due to its broad activity on plasma membrane and secreted proteins (Fan et al., 2023; Wang et al., 2021; Wu et al., 2023; Xu X. et al., 2025). To directly test the contribution of ST6GAL1 to AMA toxicity, we generated ST6GAL1-knockdown HAP1 cells. The successful knockdown was confirmed both at the transcript level (Figure 6B) and by a pronounced reduction in Cy5-SNA lectin staining, indicating loss of α2,6-sialylation at the cell surface (Figures 6C,D).

The shNT and shST6GAL1 cell lines exhibited similar baseline viability under untreated conditions. However, ST6GAL1 knockdown significantly attenuated AMA-induced cytotoxicity compared with control cells (Figure 6E), indicating a functional role of ST6GAL1-mediated sialylation in AMA sensitivity. We reasoned that the observed resistance could stem from impaired cellular uptake of the toxin. The intracellular AMA content was quantified using an established HPLC assay. The HPLC system exhibited a detection limit of 0.05 μM for AMA. The calibration curve demonstrated a strong linear relationship between AMA concentration (0.05–5 μM) and peak area, with R^2^ = 0.99998 (Supplementary Figure S2). All experimental samples fell within this linear range. Consistent with this hypothesis, HPLC quantification revealed markedly lower intracellular AMA accumulation in ST6GAL1-deficient cells relative to counterparts (Figures 6F,G). These results identify ST6GAL1-mediated sialylation as a key determinant of AMA entry into cells.

**FIGURE 7:**
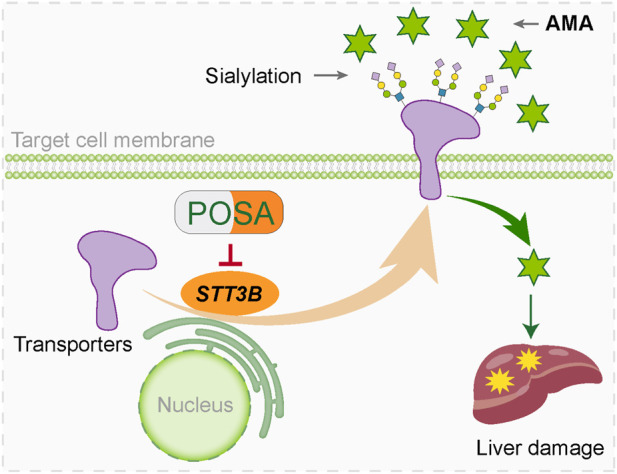
Proposed mechanism underlying the protective effect of posaconazole against AMA toxicity. Posaconazole potentially inhibits STT3B-mediated N-glycosylation, resulting in reduced terminal sialylation of cell-surface transporters. This reduction in sialylation decreases AMA uptake, thereby alleviating its intracellular toxicity and attenuating hepatocyte injury.

Collectively, our genetic and pharmacological data support a mechanism in which posaconazole potential inhibits the oligosaccharyltransferase STT3B, disrupts N-glycan biosynthesis and subsequent terminal sialylation. The resulting reduction in cell-surface sialylation decreases AMA uptake, thereby attenuating its intracellular toxicity and protecting hepatocytes from AMA-induce injury (Figure 7).

## 4 Discussion

Amatoxin poisoning represents a critical unmet medical need, with AMA being the primary hepatotoxic agent responsible for fatal outcomes. Despite the well-established role of RNA polymerase II inhibition in its pathogenesis, current clinical management remains supportive, underscoring the necessity to identify novel therapeutic targets and antidotes. Here, through a targeted drug repurposing strategy, we identify the antifungal agent posaconazole as a potent countermeasure against AMA poisoning. We demonstrate that posaconazole confers significant protection *in vitro* and *in vivo* and unveil a previously unrecognized mechanism of action centered on the disruption of STT3B-dependent N-glycosylation, which in turn impairs toxin uptake via reduced surface sialylation.

Our findings reposition posaconazole from a conventional antifungal agent to a host-directed therapeutic that targets the glycosylation pathways essential for AMA toxicity. Posaconazole significantly improved survival and mitigated hepatic and renal injury in preclinical models, even when administered after toxin exposure, a clinically relevant scenario of delayed presentation. Its comparable efficacy to ICG, coupled with a more favorable pharmacokinetic profile, supporting its potential as a therapeutic candidate for amatoxin poisoning. A limitation of the present study is that only intravenous posaconazole was evaluated. Intravenous administration was selected to ensure rapid and consistent drug delivery during acute intoxication. Although oral posaconazole is widely used clinically and may also provide protection if sufficient systemic and hepatic exposure can be achieved, delayed absorption could limit its utility in acute amatoxin poisoning, where timely intervention is critical. Future studies should evaluate the pharmacokinetics and therapeutic efficacy of oral posaconazole in this setting.

The mechanistic insights from this study extend beyond therapeutic discovery. While AMA toxicity has traditionally been attributed to RNA polymerase II inhibition, our findings identify host glycosylation as a key determinant of AMA cytotoxicity. By integrating computational docking, genetic perturbation, and metabolic labeling approaches, we provide evidence that posaconazole potential inhibits STT3B-dependent N-glycosylation. We further identify ST6GAL1-mediated α2,6-sialylation as a critical facilitator of AMA uptake. Consistent with this model, genetic ablation of ST6GAL1 recapitulated the effects of posaconazole on AMA resistance, supporting the role for sialylated glycoproteins in toxin internalization.

This discovery opens several compelling avenues for future research. The identity of the specific sialylated glycoprotein(s) that mediate AMA uptake remains to be elucidated. It is plausible that these could involve cell-surface transporters or receptors that facilitate the toxin’s journey into the cytosol. Furthermore, given the pleiotropic roles of sialylation in immune recognition and sterile inflammation, it would be valuable to investigate whether the reduction in sialylation contributes to the attenuated macrophage and neutrophil infiltration observed in our *in vivo* model, thereby adding an immunomodulatory layer to its protective mechanism.

In conclusion, our work provides a multifaceted advance in the field of amatoxin toxicology. We not only nominate posaconazole, an FDA-approved drug, as a readily repurposable antidote but also decipher a novel glycosylation-dependent pathway that is fundamental to AMA hepatotoxicity. These findings challenge the singular focus on nuclear events and establish a new paradigm for combating hepatotoxic insults by targeting the host’s glycoproteome. This strategy may hold promise for the treatment of other xenobiotic injuries where host factors mediate tissue-specific vulnerability.

## Data Availability

The original contributions presented in the study are included in the article/Supplementary Material, further inquiries can be directed to the corresponding authors.
