# Supplementary material for: Posaconazole mitigates α-amanitin-induced liver injury by inhibiting STT3B-mediated glycosylation and cellular uptake
Source: Front Pharmacol. 2026 Jul 10;17:1864484. doi: 10.3389/fphar.2026.1864484 (PMC13395608; doi:10.3389/fphar.2026.1864484)
Supplement: Supplementary file 1 [file Supplementaryfile1.docx]

Supplementary Material

**Posaconazole mitigates α-amanitin-induced liver injury by inhibiting STT3B-mediated glycosylation and cellular uptake**

Bei Wang, Chenwei Wang, Yizhi Zhang, Yingjie Yu, Jiahao Yuan, Pan Chen, Jie Chen, Yubo Tang, Qiao-Ping Wang, Ke-Jing Tang


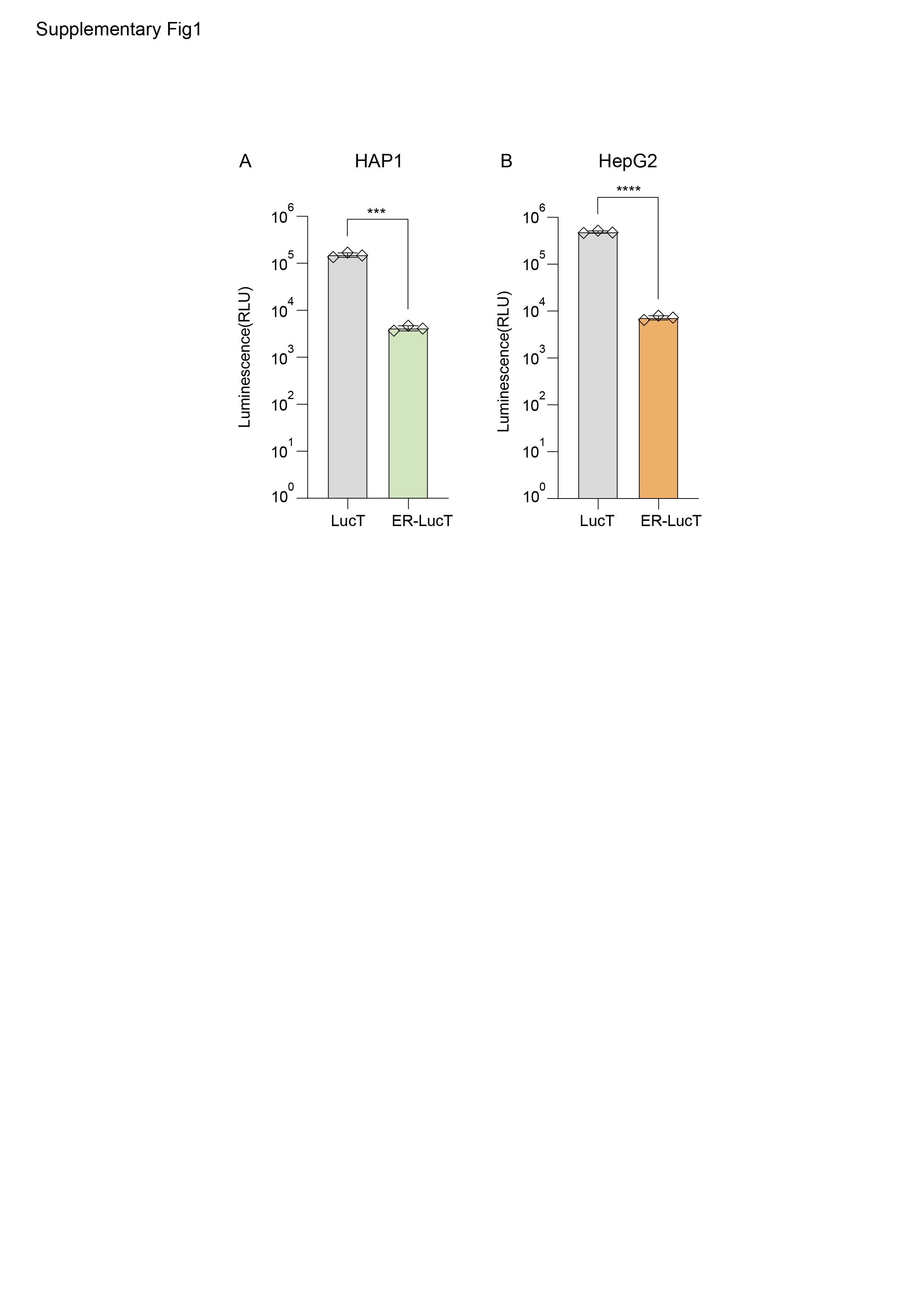


**Figure S1. N-glycosylation of Luc activity reduced by the addition of ER sequence.**

Decreased luminescence reflects the success glycosylation of ER-Luc in HAP1(**A**) and HepG2 (**B**) cells (n = 3 biological replicates). ^***^*p* = 0.0001, ^****^*p* < 0.0001. The statistics were assessed using unpaired t test.


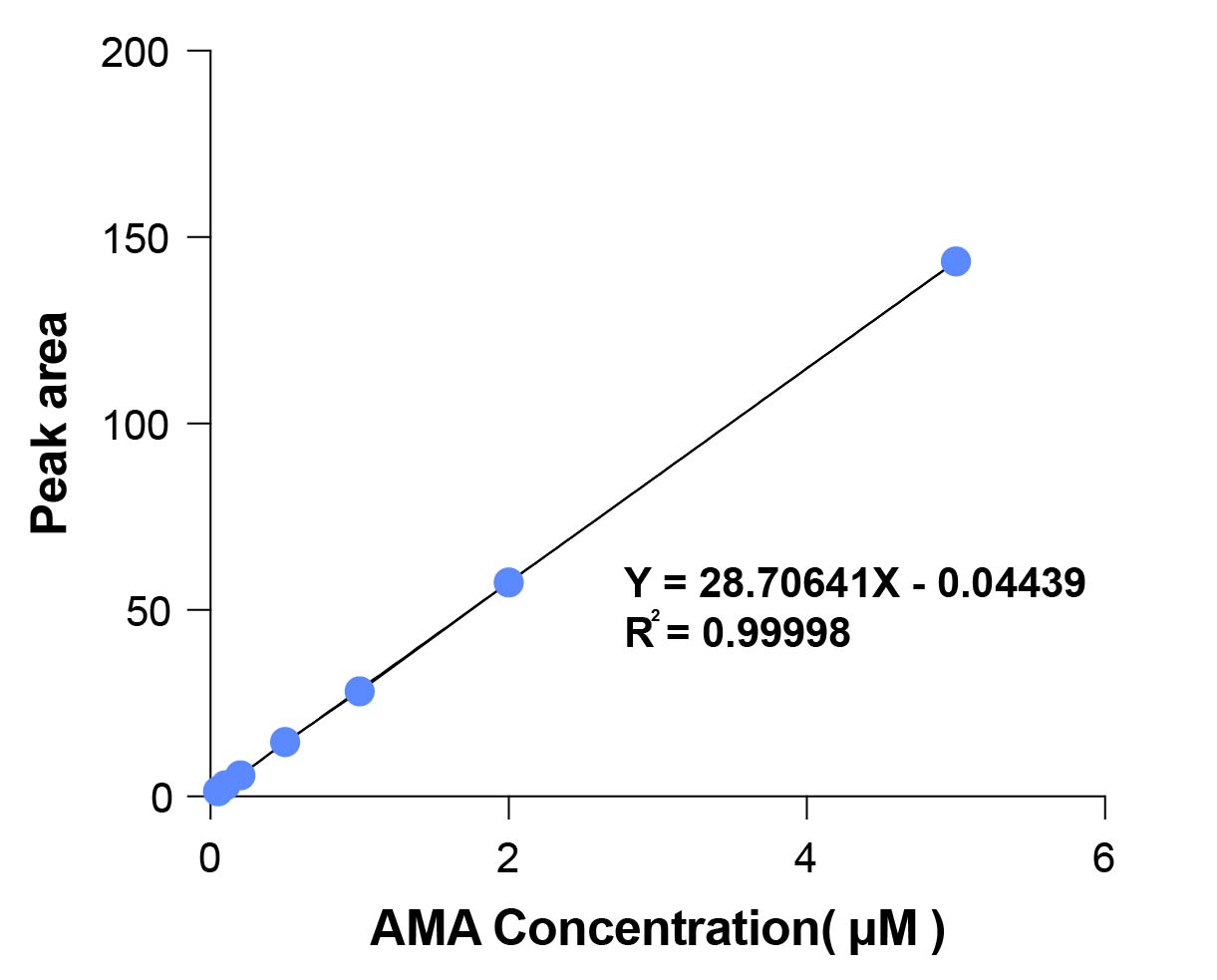


**Figure S2. The calibration curve of AMA concentration and peak area.** The calibration curve showed a good linear relationship between AMA concentration (0.05 - 5 μM) and peak area, with R^2^ = 0.99998

**Table S1. The various sequences used in this study.**

| Sequences used for gene validation | |
| --- | --- |
| shST6GAL1#1 | CGTGTGCTACTACTACCAGAA |
| shST6GAL1#2 | GCGCTTCCTCAAAGACAGTTT |
| Real-time RT-PCR primer | |
| ST6GAL1-F | CCCCAATCAGCCCTTTTACATCCTC |
| ST6GAL1-R | CCTGGTCACACAGCGTCATCATG |
| GAPDH-F | GGAGCGAGATCCCTCCAAAAT |
| GAPDH-R | GGCTGTTGTCATACTTCTCATGG |
